# Evaluation of eLIFT for Non-invasive Assessment of Liver fibrosis and Cirrhosis in Patients with Chronic Hepatitis B Virus Infection

**DOI:** 10.1038/s41598-017-05718-x

**Published:** 2017-07-14

**Authors:** Qiang Li, Chuan Lu, Weixia Li, Yuxian Huang, Liang Chen

**Affiliations:** 1 Department of Hepatitis, Shanghai Public Health Clinical Center, Fudan University, Shanghai, 201508 China; 2 Department of Infectious Diseases, Huashan Hospital, Fudan University, Shanghai, 200040 China

## Abstract

Recently, the easy Liver Fibrosis Test (eLIFT), a sum of points attributed to age, gender, gamma-glutamyl transpeptidase, aspartate transaminase, platelets, and prothrombin time, was developed for diagnosing advanced fibrosis and cirrhosis in chronic liver disease. We aimed to evaluate the performance of eLIFT to predict liver fibrosis and cirrhosis in patients with chronic hepatitis B (CHB). Histologic and laboratory data of 747 CHB patients were analyzed. The performance of eLIFT for diagnosing liver fibrosis and cirrhosis was compared with that of aspartate transaminase to platelet ratio index (APRI) and fibrosis index based on the 4 factors (FIB-4). To predict advanced fibrosis, the AUROC of eLIFT was comparable with that of APRI (0.66 *vs* 0.71, *p* = 0.095) and FIB-4 (0.66 *vs* 0.67, *p* = 0.612). To predict severe fibrosis, the AUROC of eLIFT was lower than that of APRI (0.65 *vs* 0.83, *p* < 0.001) and FIB-4 (0.65 *vs* 0.82, *p* < 0.001). To predict cirrhosis, the AUROC of eLIFT was also lower than that of APRI (0.64 *vs* 0.85, *p* = 0.001) and FIB-4 (0.64 vs 0.76, *p* = 0.033). The eLIFT is not a good non-invasive test for the diagnosis of liver fibrosis and cirrhosis in CHB patients.

## Introduction

Chronic hepatitis B virus (HBV) infection is very common: worldwide, 240 million have chronic HBV infection^1^. A recent study from China showed that HBV prevalence in men in rural China has changed from highly endemic into intermediate endemic in the past two decades, but the absolute number of HBV-infected men and the susceptible population is still large^2^. Another recent study from China also showed that the hepatitis B surface antigen (HBsAg) positive rate was 6.1%^3^. Chronic hepatitis B (CHB) can lead to a progressive accumulation of liver fibrosis which progressively evolves to cirrhosis, hepatocellular carcinoma (HCC), liver failure, and death^4^. Both the prognosis and the management of CHB patients are closely linked to the level of liver fibrosis. In CHB patients, the clinically relevant endpoints are: (1) detection of advanced fibrosis, which indicates that patients should receive antiviral treatment immediately to prevent further progression to cirrhosis and its complications; (2) detection of cirrhosis, which indicates that patients should not only potentially be treated for longer duration but also monitored for complications related to large esophageal varices and regularly screened for HCC^1^.

Liver biopsy is the reference procedure for liver fibrosis evaluation; however, limitations of this procedure include invasive nature, cost, and risk of serious complications. These limitations of liver biopsy make it unsuitable as first-line test to screen liver fibrosis in CHB patients. Recently, assessments of liver fibrosis by non-invasive tests have been developed. The FibroScan has been used for the non-invasive evaluation of liver fibrosis in clinical practice, and provides an exciting alternative to liver biopsy^5^. However, the FibroScan device is expensive and only accessible in specialized centers in developing countries, and not all CHB patients can be referred to the specialized centers. Therefore, the development of tests that can be easily used by all physicians to detect liver fibrosis and cirrhosis is greatly urgent.

In January 2017, Boursier *et al*. developed a novel algorithm-the easy Liver Fibrosis Test (eLIFT), a sum of points attributed to age, gender, gamma-glutamyl transpeptidase (GGT), aspartate transaminase (AST), platelet count, and prothrombin time, for the diagnosis of advanced fibrosis and cirrhosis in the training set of 2503 patients with chronic liver disease (CLD)^6^. In the validation set of 1251 patients with CLD, eLIFT and FIB4 had the same sensitivity (78.0% *vs* 76.6%, *p* = 0.47) but eLIFT gave less false-positive results (53.8% *vs* 82.0%, *p* < 0.001), and was thus more suitable as screening test^6^. In a word, the eLIFT was identified as a novel noninvasive algorithm, which was able to estimate advanced fibrosis and cirrhosis in CLD patients. However, in the study by Boursier *et al*., the main causes of CLD were chronic hepatitis C (45.5%) and nonalcoholic fatty liver disease (NAFLD) (34.2%), and the prevalence of CHB was only 3.2%^6^. As the authors conclude, the study is limited by the presence of a subgroup with undetermined diagnosis, and further works will be needed to see if eLIFT can provide a precise diagnosis in the subgroup of CHB patients^6^.

At present, there is a lack of data about the performance of eLIFT for the diagnosis of liver fibrosis and cirrhosis in CHB patients, and clinical research is needed urgently to see if eLIFT can provide a precise diagnosis of liver fibrosis and cirrhosis in CHB patients. In this study, we evaluated the diagnostic performance of eLIFT for advanced fibrosis, severe fibrosis, and cirrhosis, and compared with that of APRI and FIB-4 in 747 CHB patients.

## Patients and Methods

### Patients

**Figure 1:**
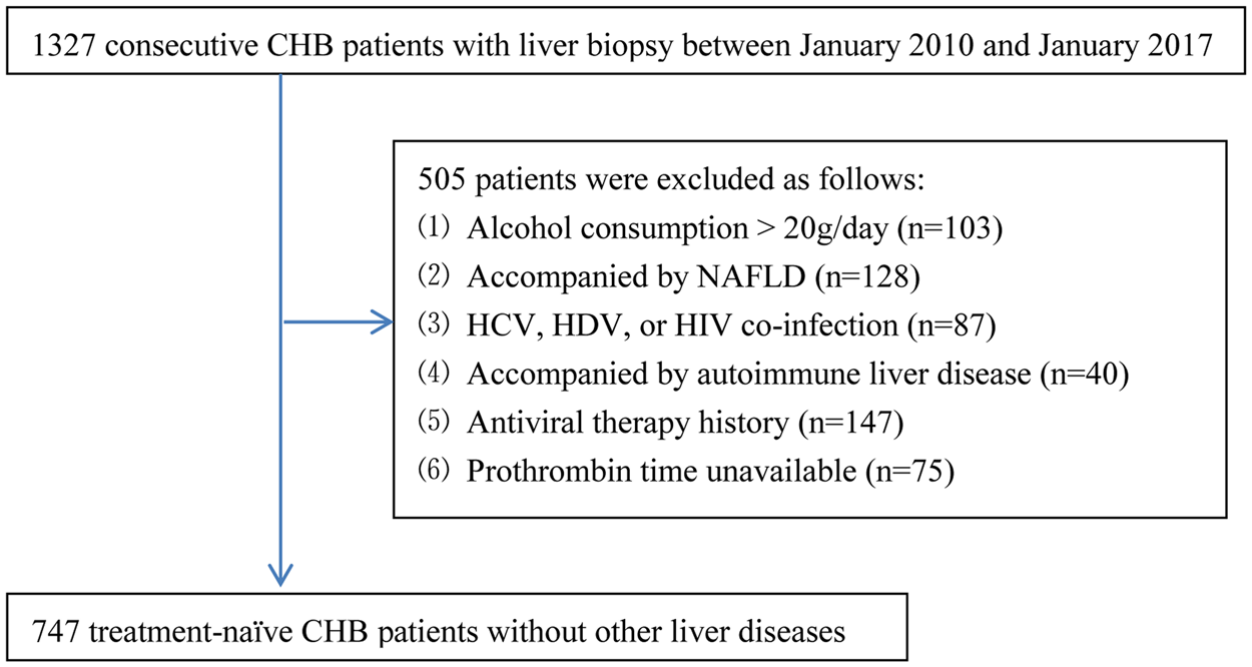
Flow diagram of the study population. CHB, chronic hepatitis B; NAFLD, non-alcoholic fatty liver disease; HCV, hepatitis C virus; HDV, hepatitis D virus; HIV, human immunodeficiency virus.

Thirteen hundred and twenty-seven consecutive CHB patients who underwent liver biopsies and routine laboratory tests in Shanghai Public Health Clinical Center, Shanghai, China, between January 2010 and January 2017 were retrospectively screened. CHB was defined as the persistent presence of HBsAg for more than six months^7^. Exclusive criteria: (1) alcohol consumption ≥20 g/day (n = 103); (2) coexistence with NAFLD defined as the presence of more than 5% steatosis of hepatocytes and the clinical records indicated that the patient was felt to either be totally abstinent or to consume less than approximately 20 g of alcohol daily (n = 128); (3) hepatitis C virus (HCV), hepatitis D virus (HDV), or HIV co-infection (n = 87); (4) coexistence with autoimmune liver disease (n = 40); (5) the history of antiviral therapy (n = 147); (6) prothrombin time unavailable (n = 75). Finally, seven hundred and forty-seven CHB patients were included. Figure 1 summarized the flow diagram of the enrolled patients.

All patients signed the informed consent before liver biopsy, and all clinical procedures were in accordance with the Helsinki declaration. The ethics committee of Shanghai Public Health Clinical Center approved the study protocol, and experiments, including any relevant details. All experiments were performed in accordance with relevant guidelines and regulations.

### Liver Biopsy and Histological Score

Liver biopsy was performed under ultrasound localization in all enrolled patients, and a minimum of 15 mm of liver tissue with at least six portal tracts is considered sufficient for histological scoring. Histological scoring was performed by two liver pathologists who were blinded for the data of non-invasive tests. In case of discrepancies, slides were reviewed by a third senior expert specialized in hepatology. The METAVIR scoring system was adopted as the gold standard of liver fibrosis, which was classified into five stages: F0, no fibrosis; F1, portal fibrosis without septa; F2, portal fibrosis with rare septa; F3, numerous septa without cirrhosis; and F4, cirrhosis^8^. For this study, we defined none/mild fibrosis as METAVIR F0-1, advanced fibrosis as METAVIR F2-4, severe fibrosis as METAVIR F3-4, and cirrhosis as METAVIR F4.

### Blood Fibrosis Tests

The data available enabled the calculation of three blood fibrosis tests according to published formulas as follows:

1. eLIFT is the sum of age, gender, GGT, AST, platelet count, and prothrombin time^6^: age (years): <40 = 0, ≥40 = 3; gender: female = 0, male = 1; AST (IU/L): <35 = 0, 35–69 = 2, ≥70 = 4; GGT (IU/L): <35 = 0, 35–89 = 1, ≥90 = 2; platelet count (10^9^/L): ≥250 = 0, 170–249 = 1, <170 = 4; prothrombin time (%): ≥97 = 0, 84–96 = 2, <84 = 4;
2. APRI^9^ = (AST/ULN of AST)/platelet count ×100;
3. FIB-4^10^ = (age ×AST)/(platelet count ×(ALT)^1/2^).

### Statistics

Normality tests of baseline data were performed by Kolmogorov-Smirnov test. The baseline characteristics of patients are presented as follows: normal distribution data as mean ± standard deviation, non-normal distribution continuous data as median (interquartile range), and categorical variables as number (percentage). The correlations between noninvasive markers and METAVIR fibrosis scores were analyzed using Spearman test. The ROC curve analysis was performed to evaluate the performances of non-invasive tests for the diagnosis of advanced fibrosis, severe fibrosis, and cirrhosis, respectively. Two sets of cut-offs were calculated: (1) maximizing Youden’s index (sensitivity + specificity−1), which is an optimized balance between sensitivity and specificity, or (2) obtaining a sensitivity of at least 90%. Diagnostic accuracy was evaluated by sensitivity, specificity, positive predictive value (PPV), and negative predictive value (NPV), positive likelihood ratio (+LR), and negative likelihood ratio (−LR). All significance tests were two-tailed, and *p* < 0.05 was considered statistically significant. All statistical analyses were carried out using the SPSS software version 15.0 (SPSS Inc. Chicago, Illinois, USA) and MedCalc Software version 16.1 (MedCalc Software bvba, Ostend, Belgium).

## Results

### Baseline Data

Baseline characteristics of enrolled patients were presented in Table 1. The median age was 36 years, and mostly male (65.2%), and HBeAg positive (67.3%). The median HBV DNA, ALT, AST, GGT, platelet count, and prothrombin time was 6.6 log10 copies/ml (IQR = 4.2–7.6), 42 IU/L (IQR = 28–62), 29 IU/L (IQR = 22–39), 24 IU/L (IQR = 15–44), 177 × 10^9^/L (IQR = 159–215), and 88% (IQR = 82%–96%), respectively. The median APRI and FIB-4 was 0.40 (IQR = 0.28–0.61) and 0.82 (IQR = 0.61–1.19), respectively. The METAVIR inflammation stage was distributed as follows: A0 = 114 (15.3%); A1 = 349 (46.7%); A2 = 186 (24.9%); and A3 = 98 (13.1%). The METAVIR fibrosis stage was distributed as follows: F0 = 74 (9.9%); F1 = 438 (58.6%); F2 = 106 (14.2%); F3 = 54 (7.2%); and F4 = 75 (10.1%). Among 747 patients, 235 (31.5%), 129 (17.3%), and 75 (10.1%) were classified as having advanced fibrosis, severe fibrosis, and cirrhosis, respectively.

**Table 1:** Baseline characteristics of the study population.

Characteristics	All (n = 747)	F0-1 (n = 512)	F2-4 (n = 235)	P value
Age (years)	36 (29–44)	34 (28–42)	39 (31–49)	<0.001
Male gender, n (%)	487 (65.2%)	322 (62.9%)	165 (70.2%)	0.051
HBeAg positive, n (%)	503 (67.3%)	343 (67.0%)	160 (68.1%)	0.767
HBV DNA (log10 copies/ml)	6.6 (4.2–7.6)	6.5 (3.9–7.4)	6.6 (4.3–7.7)	0.875
ALT (IU/L)	42 (28–62)	41 (27–60)	44 (30–71)	0.067
AST (IU/L)	29 (22–39)	27 (22–36)	31 (24–46)	<0.001
GGT (IU/L)	24 (15–44)	18 (13–33)	42 (25–70)	<0.001
Platelet count (10^9^/L)	177 (159–215)	194 (170–242)	145 (136–168)	<0.001
Prothrombin time (%)	88 (82–96)	92 (86–99)	81 (73–88)	<0.001
APRI	0.40 (0.28–0.61)	0.34 (0.24–0.57)	0.53 (0.33–0.70)	<0.001
FIB-4	0.82 (0.61–1.19)	0.77 (0.59–1.15)	0.94 (0.66–1.27)	<0.001
METAVIR Inflammation stage				
A0	114 (15.3%)	80 (15.6%)	34 (14.5%)	0.683
A1	349 (46.7%)	246 (48.0%)	103 (43.8%)	0.283
A2	186 (24.9%)	117 (22.9%)	69 (29.4%)	0.056
A3	98 (13.1%)	69 (13.5%)	29 (12.3%)	0.669
METAVIR fibrosis stage				
F0	74 (9.9%)	74 (9.9%)	0	
F1	438 (58.6%)	438 (58.6%)	0	
F2	106 (14.2%)	0	106 (14.2%)	
F3	54 (7.2%)	0	54 (7.2%)	
F4	75 (10.1%)	0	75 (10.1%)	

ALT, alanine transaminase; AST, aspartate transaminase; GGT, gamma-glutamyl transpeptidase; APRI, AST-to-platelet ratio index; FIB-4, fibrosis index based on the 4 factors; F0-1, non-advanced fibrosis; F2-4, advanced fibrosis.

Patients with advanced fibrosis had higher age (39 *vs* 34 years, *p* < 0.001), AST (31 *vs* 27 IU/L, *p* < 0.001), GGT (42 *vs* 18 IU/L, *p* < 0.001), APRI (0.53 *vs* 0.34, *p* < 0.001), and FIB-4 (0.94 *vs* 0.77, *p* < 0.001) levels, but lower platelet count (145 *vs* 194 × 10^9^/L, *p* < 0.001) and prothrombin time (81% *vs* 92%, *p* < 0.001) than patients with none/mild fibrosis. No significantly differences were seen in sex, HBeAg status, HBV DNA levels, ALT levels, and METAVIR inflammation stages between patients with advanced fibrosis and patients with none/mild fibrosis (Table 1).

### Correlations between Noninvasive Markers and METAVIR Fibrosis Stages

**Figure 2:**
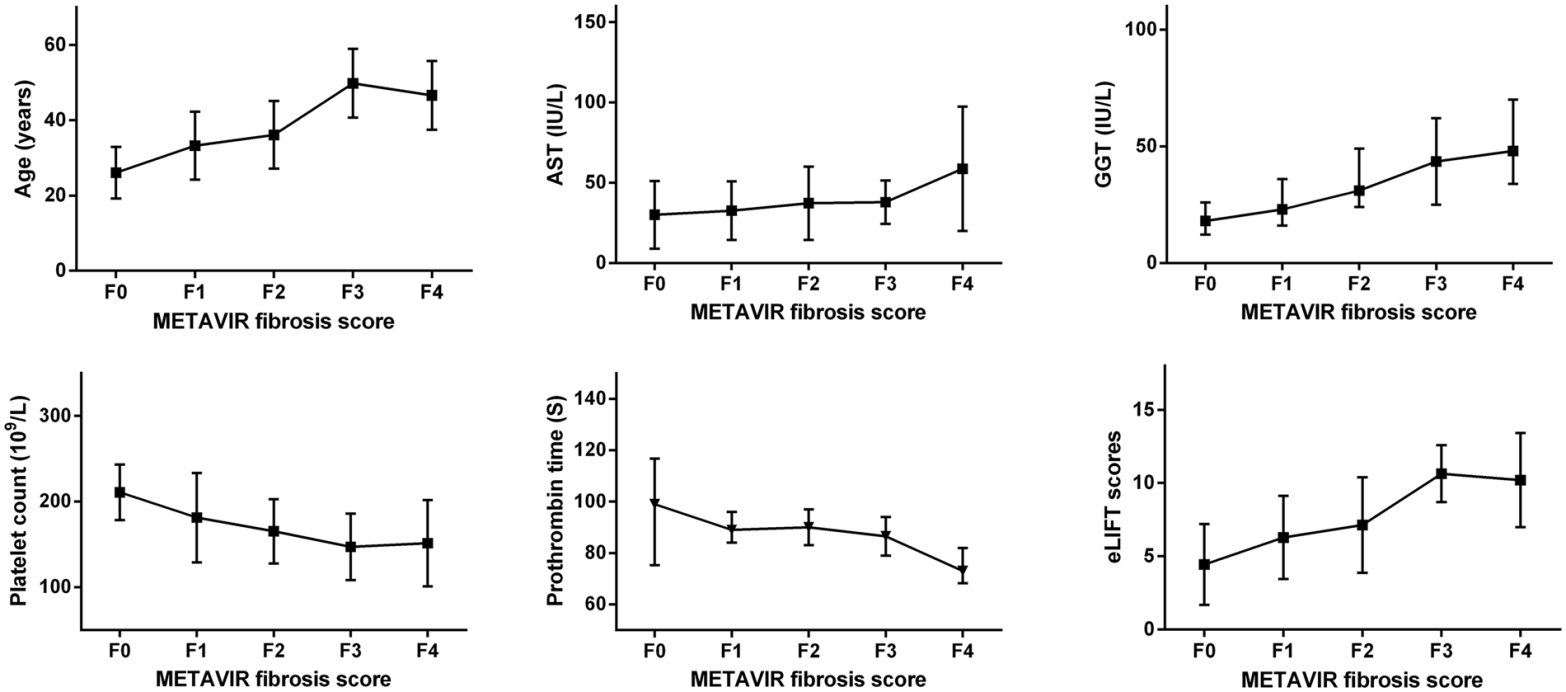
Association between noninvasive markers and METAVIR fibrosis stages. AST, aspartate transaminase; GGT, gamma-glutamyl transpeptidase; eLIFT, the easy Liver Fibrosis Test. The METAVIR fibrosis stages had positive correlation with age, AST, GGT, and eLIFT scores; but negative correlation with platelet count and prothrombin time. The higher median age, AST, GGT, and eLIFT scores, but lower platelet count levels and prothrombin time with increasing METAVIR fibrosis stages.

The correlations between noninvasive markers and METAVIR fibrosis stages were showed in Table 2. The age (Spearman’s correlation coefficient r = 0.26, *p* < 0.001), male (r = 0.24, *p* < 0.001), AST (r = 0.19, *p* < 0.001), and GGT (r = 0.45, *p* < 0.001) had positive correlation with METAVIR fibrosis stages; and platelet count (r = −0.40, *p* < 0.001) and prothrombin time (r = −0.33, *p* < 0.001) had negative correlation with METAVIR fibrosis stages. The METAVIR fibrosis stages were positive correlated with eLIFT scores (r = 0.49, *p* < 0.001), with higher correlation coefficient than any maker alone. The association between METAVIR fibrosis stages and noninvasive markers was presented in Fig. 2.

**Table 2:** Correlations between noninvasive markers and METAVIR fibrosis stages.

Variables	Spearman’s r	P value
Age (years)	0.26	**<0.001**
Male	0.24	**<0.001**
AST (IU/L)	0.19	**<0.001**
GGT (IU/L)	0.45	**<0.001**
Platelet count (10^9^/L)	−0.40	**<0.001**
Prothrombin time (%)	−0.33	**<0.001**
eLIFT	0.49	**<0.001**

AST, aspartate transaminase; GGT, gamma-glutamyl transpeptidase; eLIFT, the easy Liver Fibrosis Test; Spearman’s r, correlation coefficient.

### Diagnostic Performance of eLIFT for Liver Fibrosis and Cirrhosis

**Figure 3:**
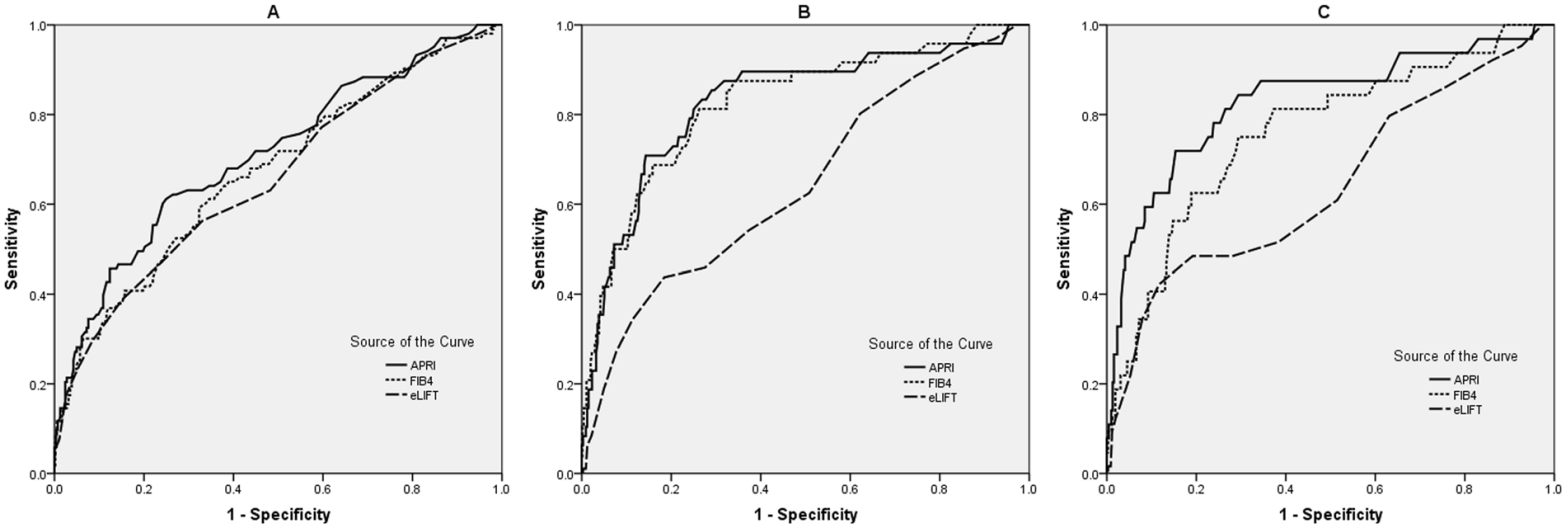
ROC curves for significant fibrosis (**A**), severe fibrosis (**B**), and cirrhosis (**C**) APRI, aspartate transaminase to platelet ratio index; FIB-4, fibrosis index based on the 4 factors; eLIFT, the easy Liver Fibrosis Test; The AUROC of eLIFT was comparable with that of APRI and FIB-4 to predict advanced fibrosis (*p* > 0.05), but lower than that of APRI and FIB-4 to predict severe fibrosis and cirrhosis (*p* < 0.05).

The diagnostic performances of eLIFT, APRI, and FIB-4 for liver fibrosis and cirrhosis were presented in Table 3, and the ROC curves were showed in Fig. 3. To predict advanced fibrosis, the AUROC of eLIFT was comparable with that of APRI (0.66 *vs* 0.71, *p* = 0.095) and FIB-4 (0.66 *vs* 0.67, *p* = 0.612). To predict severe fibrosis, the AUROC of eLIFT was lower than that of APRI (0.65 *vs* 0.83, *p* < 0.001) and FIB-4 (0.65 *vs* 0.82, *p* < 0.001). To predict cirrhosis, the AUROC of eLIFT was lower than that of APRI (0.64 *vs* 0.85, *p* = 0.001) and FIB-4 (0.64 *vs* 0.76, *p* = 0.033).

**Table 3:** Diagnostic performances of noninvasive tests for liver fibrosis and cirrhosis.

	Advanced fibrosis		Severe fibrosis		Cirrhosis	
	AUROC	(95% CI)	AUROC	(95% CI)	AUROC	(95% CI)
APRI	0.71	(0.68–0.74)	0. 83	(0.80–0.85)	0. 85	(0.82–0.87)
FIB-4	0.67	(0.64–0.71)	0.82	(0.80–0.84)	0.76	(0.73–0.79)
eLIFT	0.66	(0.62–0.69)	0.65	(0.61–0.68)	0.64	(0.61–0.68)
Comparing AUROC						
eLIFT and APRI	*p* = 0.095		***p*** < ***0.001***		***p*** ** = 0.001**	
eLIFT and FIB-4	*p* = 0.612		***p*** < ***0.001***		***p = 0.033***	
APRI and FIB-4	***p*** ** = 0.03**		*p* = 0.91		***p*** ** = 0.005**	

APRI, aspartate transaminase to platelet ratio index; FIB-4, fibrosis index based on the 4 factors; eLIFT, the easy Liver Fibrosis Test; AUROC, the area under the receiver operating characteristic curve; CI, confidence interval.

### Diagnostic thresholds of eLIFT for liver fibrosis and cirrhosis

Diagnostic thresholds of eLIFT for liver fibrosis and cirrhosis were presented in Table 4. By maximizing Youden’s index, the optimal cut-off of eLIFT was 9, refs 9 and 10, respectively, for the diagnosis of advanced fibrosis (the sensitivity, specificity, PPV, and NPV was 39%, 85%, 49%, and 79%, respectively), severe fibrosis (the sensitivity, specificity, PPV, and NPV were 44%, 82%, 26%, and 91%, respectively), and cirrhosis (the sensitivity, specificity, PPV, and NPV were 42%, 88%, 25%, and 94%, respectively). By obtaining a sensitivity of at least 90%, the cut-off of eLIFT was 3, 3, and 5, respectively, for the diagnosis of advanced fibrosis (the sensitivity, specificity, PPV, and NPV were 94%, 16%, 30%, and 87%, respectively), severe fibrosis (the sensitivity, specificity, PPV, and NPV were 95%, 14%, 14%, and 95%, respectively), and cirrhosis (the sensitivity, specificity, PPV, and NPV were 92%, 14%, 9%, and 95%, respectively).

**Table 4:** Diagnostic thresholds of eLIFT for liver fibrosis and cirrhosis.

	Cut-offs	Se,%	Sp,%	PPV,%	NPV, %	+LR	−LR
Advanced fibrosis	9*	39	85	49	79	2.60	0.72
	3**	94	16	30	87	1.11	0.38
Severe fibrosis	9*	44	82	26	91	2.37	0.69
	3**	95	14	14	95	1.11	0.36
Cirrhosis	10*	42	88	25	94	3.50	0.65
	5**	92	14	9	95	1.07	0.57

eLIFT, the easy Liver Fibrosis Test; Se, sensitivity; Sp, specificity; PPV, positive predictive value; NPV, negative predictive value; +LR, positive likelihood ratio; −LR, negative likelihood ratio; Cut-offs* were obtained by maximizing Youden index (sensitivity + specificity−1); Cut-offs** were established by obtaining a sensitivity of at least 90%.

## Discussion

The early management of CHB patients had been recommended by all international guidelines^1, 7, 11^. However, a systematic review of 118 studies showed that two thirds of patients with cirrhosis were diagnosed belatedly when complications appeared^12^. This demonstrates an urgent need for non-invasive tests to screen patients with advanced fibrosis and cirrhosis, who require antiviral management immediately for preventing the disease progress. In this setting, the non-invasive diagnosis of liver fibrosis has been a hot field of research. A study by Jia *et al*. showed that liver elastometry got high AUROCs of 0.82, 0.88, and 0.90 for the diagnoses of advanced fibrosis, severe fibrosis, and cirrhosis, respectively^13^. Recently, the study by Shi *et al*. showed that magnetic resonance elastography technique had almost perfect AUROCs of 0.97 and 0.98 for the diagnoses of advanced fibrosis and cirrhosis, respectively^14^. Although liver elastometry and magnetic resonance elastography have been increasingly recognized as the excellent tools for the diagnosis of liver fibrosis, they are currently available only in specialized tertiary centers because of the devices were expensive. Simple, inexpensive, and accurate fibrosis tests are urgently needed for screening CHB patients for liver fibrosis and cirrhosis.

The blood fibrosis tests such as APRI and FIB-4 have been used widely because of the noninvasive procedure, comprising only inexpensive laboratory tests, and available in primary care. The eLIFT is a new, user-friendly, at-a-glance fibrosis test available to all physicians, whether specialized in hepatology or not, who manage CLD patients in their daily clinical practice, as it is based on parameters commonly assessed in CLD^6^. Compared to APRI and FIB-4, the eLIFT has two main advantages^6^. First, while the APRI and FIB4 need a computer for calculation, eLIFT is very easily calculated at-a-glance and in one’s head, which makes eLIFT easier and faster to use in clinical practice than APRI and FIB4^6^. More importantly, the eLIFT gave less false-positive results in the validation set of 1251 patients with CLD (53.8% *vs* 82.0%, *p* < 0.001), and was thus more suitable as screening test^6^.

Given that the eLIFT was developed in patients with CLD-the vast majority of them were HCV (45.5%) and NAFLD (34.2%) and just a small number of them were HBV (3.2%), it would be necessary to evaluate the diagnostic performance of eLIFT for liver fibrosis and cirrhosis in CHB patients before it can be popularized widely in this populations. The eLIFT needs further evaluation in subgroup of CLD because of the different pathogenesis, patterns of fibrosis, and patterns of regression have been observed in different causes of CLD^15^. In this retrospective study of 747 CHB patients, we observed that the eLIFT did not get higher performance than APRI and FIB-4, which have been recommended by the WHO HBV guidelines^16^.

The AUROCs of eLIFT for diagnosing advanced fibrosis and cirrhosis were unsatisfactory (0.66 and 0.64, respectively). The optimal cut-off values for excluding and confirming advanced fibrosis were 3 and 9, respectively: and these cut-offs were not useful either for excluding or confirming presence of advanced fibrosis (negative likelihood ratio 0.38 for cut-off 3 and positive likelihood ratio 2.60 for cut-off 9). Based on 31.5% pre-test probability of advanced fibrosis, the post-test probability of having advanced fibrosis increased to only 49% with positive test (eLIFT ≥ 9). Similarly, the optimal cut-offs for excluding and confirming cirrhosis were 5 and 10, respectively: and these cut-offs were also not useful either for excluding or confirming presence of cirrhosis (negative likelihood ratio 0.57 for cut-off 5 and positive likelihood ratio 3.50 for cut-off 10). Based on 10.1% pre-test probability of cirrhosis, the post-test probability of having cirrhosis increased to only 25% with positive test (eLIFT ≥ 10).

The limitations of this study were difficult to avoid. First, the retrospective design of this study might have caused selective bias^17^. Second, there was no validation of noninvasive tests such as Hepascore, Fibrotest, or Fibrometer, because these tests were all protected by patents, and some laboratory tests which are necessary for the calculations of these tests were costly and difficult-to-obtain in our hospital. Third, this study has been conducted in tertiary referral centres with a higher proportion of patients with liver fibrosis and cirrhosis than in the general population. Thus, it might produce bias when the performance of eLIFT was extrapolated to the general CHB population.

In conclusion, eLIFT, which showed application prospect in identifying advanced fibrosis and cirrhosis in CLD patients, was not a good non-invasive test for the diagnosis of liver fibrosis and cirrhosis in CHB patients. The larger sample, prospective, multi-centre studies will be necessary to validate the performance of eLIFT in CHB patients further.

### Role of the Sponsor

The funding organizations are public institutions and had no role in the design and conduct of the study; collection, management, and analysis of the data; or preparation, review, and approval of the manuscript.
